# Urban Air Pollution and Mental Stress: A Nationwide Study of University Students in China

**DOI:** 10.3389/fpubh.2021.685431

**Published:** 2021-07-02

**Authors:** Weifang Zhang, Sihui Peng, Jialu Fu, Ke Xu, Huihui Wang, Yu Jin, Tingzhong Yang, Randall R. Cottrell

**Affiliations:** ^1^The Affiliated Hospital of Stomatology, School of Stomatology, Zhejiang University School of Medicine, Key Laboratory of Oral Biomedical Research of Zhejiang Province, Hangzhou, China; ^2^School of Medicine, Jinan University, Guangzhou, China; ^3^Epidemiology Department, Emory University, Atlanta, GA, United States; ^4^Zhejiang University Educational Foundation, Hangzhou, China; ^5^Women' s Hospital/Center for Tobacco Control Research, Zhejiang University School of Medicine, Hangzhou, China; ^6^Public Health Program, School of Health and Applied Human Sciences, University of North Carolina, Wilmington, NC, United States

**Keywords:** air pollution, mental stress, public health, environmental policy, university students

## Abstract

**Background:** Studies exploring the relationship between air pollution levels and mental stress have rarely been done, and no studies have been done comparing university student mental stress levels based on regional air pollution levels.

**Objectives:** The objective of this study was to evaluate the association between air pollution and mental stress among university students.

**Methods:** Participants were 11,942 students, who were identified through a multistage survey sampling process conducted in 50 universities. Regional air pollution levels were retrieved from a national database, and mental stress was measured using a perceived stress scale. Both unadjusted and adjusted methods were utilized in the data analyses.

**Results:** Mental stress prevalence was 36.9% (95% Confidence Interval: 24.4–49.5%). The final model indicated that regional air pollution levels were positively associated with students' mental stress.

**Conclusions:** This study provided new and direct evidence of the health hazards of air pollution. The findings underscore the need to develop and implement stringent environmental protection policies, while simultaneously raising public awareness of environmental protection.

## Introduction

Air pollution is one of the main environmental problems in many countries, especially developing countries. Short-and long term exposures to air pollution can lead to increased human morbidity and mortality rates (1–4). Studies have found that exposure to air pollution was associated with biological dysregulation, including inflammation (5), and greater risk of cardiovascular disease (6) and premature mortality (7). Many experimental and observational studies also suggested that exposure to air pollution was associated with mental disorders and negative behavioral outcomes. Furthermore, some studies found that there were associations between air pollution and depression (8–10), and psychiatric emergencies (11). Psychological theories offer a window into the possible mechanisms that may link high levels of air pollution with health problems though mental stress. According to the Stimulus, Stress response and Mental health problems (SRM) model, various stimulus (S) induce stress response (R), which in turn leads to mental health problems (M) (12, 13). Some studies found that while air pollution may induce mental stress (14–16), and high perceived mental stress levels have been linked with an increased likelihood of health problems (2, 3, 17). The authors speculated that mental stress was an intermediate variable between air pollution and mental health problems (12, 13). To help confirm this speculation, however, the relationship between air pollution levels and mental stress had to be more fully explored (13). Only a few empirical studies in which air pollution was directly associated with mental stress were found (14–16).

Ecological models have emphasized that mental stress is influenced by both individual and environmental variables. However, many studies about air pollution and mental stress were limited to small, local samples that focused primarily on individual variables (14–16). Mainland China is a vast territory with much cultural diversity and large differences in economic and social development. This creates a situation where different regions have vastly different levels of air pollution. Given China's regional differences in air pollution, it would seem that region of residence might also be related to mental stress, but no studies have directly examined this variable. Region of residence is a widely accepted and stable estimate of people's exposure to air pollution (13, 18). By utilizing a large-scale, national population sample in this study, it will be possible to expand and further clarify the relationship between environmental air pollution and mental stress.

Air pollution has become a major social and public health problem in China. The purpose of this study was to investigate the relationship between regional air pollution levels and non-specific perceived stress. This study may provide supporting information for implementing more stringent environmental protection policies and raising environmental awareness among Chinese citizens.

## Methods

### Study Area and Participants

This study employed a multistage sampling design. In Stage 1 60 potential universities with medical programs were identified. These universities were part of the Bloomberg Global Initiative Project entitled “Facilitate MOH Endorsement of Tobacco Control Curriculum Implementation through Promoting Tobacco Control Curricula in Medical Schools.” The 60 participating universities represented one-third of the 180 universities that offer medical programs across China, and the universities were differentiated by regional location. Students were recruited at 50 of the 60 universities to complete the survey. Twenty-two were medical universities offering only medical programs, and 28 were comprehensive universities offering medical and non-medical programs. Non-medical students were also randomly recruited from two-thirds of all comprehensive universities.

Stage 2 of the sampling strategy involved the selection of classes within each university. All medical courses were selected in each university, and several non-medical classes (matched for academic level) were selected in each designated comprehensive university.

### Data Collection

Once an individual participant was identified and agreed to participate in the study, a structured self-administered questionnaire was administered. The questionnaire was administered during regular lectures and class sessions. It took ~30 min to complete. All responses were anonymous.

A common research protocol was utilized across all 50 universities to assure homogeneity of questionnaire administration and data collection techniques. The study was approved by the Ethics Committee at the Zhejiang University Medical Center, and verbal consent was obtained from all participants prior to data collection.

### Measures

#### Dependent Variable

Mental stress was measured by the Chinese version of the Perceived Stress Scale (CPSS) (19). This scale is comprised of 14 items that address perceptions of stress during the month prior to the survey. Items were rated on a 5-point, Likert type scale, and ranged from 0 (never) to 4 (very often). Item scores were summarized to yield a total stress score, with higher scores indicating higher perceived levels of stress. This scale has been widely used to assess stress in China, and has been shown to be an appropriate indicator of mental health status (20–23). Following previous practice, severe stress was operationalized as a score >25, which was verified by the Receiver Operating Characteristic Curve (ROC), using mental disorders gold standard. This classification has demonstrated acceptable sensitivity and specificity (19). The dependent variable in this study was a categorical variable coded dichotomously as 1 = non severe mental stress and 2 = severe mental stress.

#### Individual-Level Independent Variables

Sociodemographic questions were utilized to determine such variables as age, gender, ethnicity, father's and mother's occupation, and family income.

#### University-Level Independent Variables

University type was determined using the China university ranking system (“high level,” “middle level,” and “low level”) as established by the National Ministry of Education (24).

#### City-Level Independent Variable

Forty-five cities were included in this study. There were several independent variables that reflected potential regional variation in characteristics of the universities. The first aspect was city population, where the universities were located. Cities were categorized as having populations <5 million, from 5 million to <10 million, or 10 million and more. The second independent variable was level of economic development, as measured by per capita Gross Domestic Product (GDP) in Yuan. Both the GDP of the original province from which the students came and of the province where the university was located were measured. Categories were <40,000, from 40,000 to <50,000, 50,000 and more. The above data were obtained from the National Bureau of statistics (25). The next independent variable was the air pollution level, in the cities that the universities were located. The air pollution level refers the amount of chemical pollutants in ambient air, was measured by days of air pollution exceeding the national standard (26). This data was available from the National Bureau of statistics (27).

### Data Analysis

All data were entered into a database using Microsoft Excel. The dataset was then imported into SAS (9.3 version) for statistical analyses. Descriptive statistics were calculated for each variable. A logistic model was utilized to assess associations between the dependent and independent variables. Both unadjusted and adjusted methods were considered in the data analyses. SAS survey logistic procedures were applied in the analysis, using the university as the clustering unit, in order to account for a within-clustering correlation, attributable to the complex sample for unadjusted analysis. Associations were confirmed through application of a multilevel logistic regression model using the SAS GlIMMIX procedure (28). Three models for the three-level (individual, universities, and cities) multilevel logistic regression analyses were constructed. The first was the “null” model with random intercepts. It did not include any predictors except a constant, in assessing variation in the likelihood of an individual experiencing severe mental stress. From this model, we entered demographic and regional socioeconomic variables as fixed main effects, with severe mental stress to form the base model. From base model we entered air pollution variables to form the full model to assess the impact of air pollution on severe mental stress. The association between system variables and severe mental stress was expressed in terms of odds ratios and 95% CI < Confidence Interval>were computed. Model fit was assessed by the likelihood of a change in the −2log. We assessed the significance of the random parameter variance estimates using the Wald joint *t*-test statistic (29).

All analysis were weighted. A weighting factor was applied to each student record to adjust for variation in the probability of selection in each university (18). A non-response weight was not utilized in this study because non-response rates were very low. Since data collection took place in classes, nearly all students present completed the questionnaires.

## Results

Valid questionnaires were completed by 97.5% of the potential students, resulting in a sample of 11,942 students from 50 different universities.

Of the sample, 12.8% were <20 years of age, about 24% were either 20 or 21 years old with the remainder of the participants being more than 21 years old. Of the study sample 49.4% were male and 50.6% were female. The majority of participants (60.7%) were freshmen and sophomores and 38.5% of the participants were juniors or seniors. 94.5% of the participants were Han (see Table 1).

**Table 1:** Demographic characteristics of sample and severe mental stress prevalence.

**Group**	***N***	**% of sample**	**Prevalence**	**Unadjusted OR**
**Age (years)**				
<20	1,890	12.8	34.5	1.00
20–20.9	1,862	24.9	34.3	0.91 (0.53, 1.56)
21–21.9	2,128	23.8	44.3	1.38 (0.62, 3.01)
22–22.9	2,448	14.7	34.8	0.93 (0.47, 1.82)
23 and over	2,456	10.2	26.8	0.64 (0.28, 1.45)
**Gender**				
Male	4,249	49.4	35.6	1.00
Female	7,693	50.6	38.3	1.12 (0.58, 2.16)
**Grade**				
1–2	4,945	60.7	36.0	1.00
3–4	6,717	38.5	39.5	1.16 (0.49, 2.74)
5 and over	292	0.8	17.3	0.39 (0.16, 0.90)^*^
**Ethnicity**				
Han	11,136	94.5	36.2	1.00
Minority	806	4.2	37.9	0.90 (0.44, 1.85)
**Major**				
Medical	10,507	81.1	32.6	1.00
Others	1,435	18.9	37.9	1.26 (0.66, 2.41)
**Income in each person in family (RMB)^#^**				
<10,000	1,811	34.3	38.4	1.00
10,000–19,999	1,273	21.7	42.0	1.66 (0.85, 1.59)
20,000 and over	1,932	44.7	33.6	0.81 (0.64, 0.97)^*^
**UNIVERSITY VARIABLES**				
**Universities types**				
High level	4,283	58.9	36.9	1.00
Middle level	6,961	38.7	35.4	0.93 (0.31, 2.82)
Low level	698	2.4	64.1	3.05 (1.45, 6.44)^**^
**REGIONAL VARIABLES**				
**Original region GDP (RMB)^#^**				
<50,000	5,980	52.1	38.3	1.00
50,000–99,999	3,550	26.3	37.0	0.95 (0.31, 2.86)
100,000 and over	2,402	21.6	33.6	0.93 (0.61, 1.42)
**University city GDP (RMB)^#^**				
<50,000	4,055	13.9	31.5	1.00
50,000–99,999	6,371	61.1	37.6	1.31 (0.48, 3.59)
100,000 and over	1,516	22.9	38.9	1.38 (0.29, 6.67)
**City population (million)**				
<1	3,084	12.2	46.1	1.00
1–3,999	5,980	57.5	42.3	0.85 (0.29, 2.45)
4 and over	2,878	30.3	23.1	0.35 (0.16, 0.77)^**^
**Air pollution status**				
<15	3,002	25.7	23.2	1.00
15–25	2,140	16.5	45.5	1.57 (1.09, 2.26)^*^
≥25	6,800	57.9	40.6	1.74 (1.19, 2.56)^**^

* *P <0.05*,

** *P <0.01*.

# *Chinese currency name*.

Severe mental stress prevalence was 36.9% (95% CI: 24.4–49.5%). The unadjusted logistic analysis showed: being junior or senior status, family income, university types, and city population were associated with lower mental stress levels. Being freshmen or sophomores and more air pollution were associated with higher mental stress prevalence.

The basic multilevel logistic regression model found university type, and city population were significantly associated with students' mental stress. The full model showed that type of university, city population, and air pollution were significantly associated with the students' mental stress (see Table 2).

**Table 2:** Results of multiple level models.

**Group**	**Null Model**	**Base Model**	**Full Model**
	**OR (95% CI)**	**OR (95% CI)**	**OR (95% CI)**
**Grade**			
1–2		1.00	
3–4		1.23 (0.52, 2.89)	
5-		0.41 (0.18, 0.94)^*^	
**Universities types**			
High level		1.00	1.00
Middle level		0.93 (0.31, 2.74)	0.40 (0.15, 1.08)
Low level		3.28 (1.46, 5.35)^**^	2.22 (1.04, 3.57)^*^
**City population (million)**			
<1		1.00	1.00
1–3,999		0.83 (0.29, 2.33)	1.22 (0.24, 4.16)
4 and over		0.34 (0.16, 0.76)^**^	0.16 (0.06, 0.42)^**^
**Air pollution**			
<15			1.00
15–25			1.18 (0.28, 3.33)
≥25			6.67 (2.56, 16.7)^**^
Fixed parameters	−0.5592 (0.1821)^**^	0.7542 (0.2883)	0.8745 (0.3441)
Random parameters between universities	1.6355 (0.3425)^**^	1.5988 (0.6547)^**^	1.4998 (0.7847)^**^
Random parameters between universities cities	1.6514 (0.3352)^**^	1.6124 (0.6628)^**^	1.5083 (0.7946)^**^

* *P <0.05*,

** *P <0.01*.

**Figure 1:**
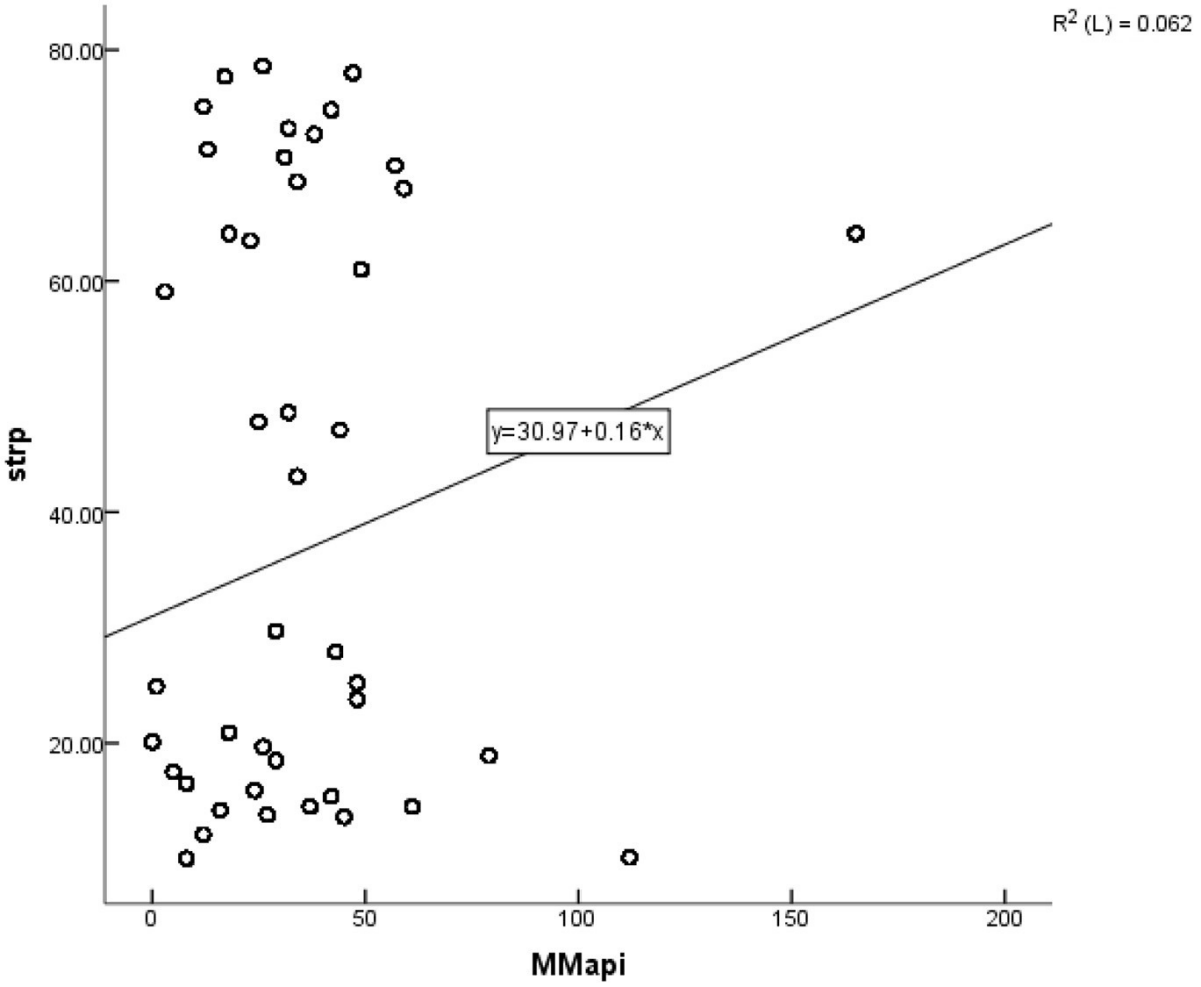
Scatter plot of the association between city-level air pollution days at each year (MMapi) and severe mental stress prevalence (strp).

Significant positive correlations were found between city-level air population time and likelihood of severe mental stress, Pearson r were 0.32452 (*p* < 0.0001). Figure 1 showed that the severe mental stress prevalence increasing with city-level air pollution days at each year increasing.

## Discussion

Based on the results of this study, 36.9% (95% CI: 24.4–49.5%) of university residents were severely stressed. This finding was very similar to stress levels reported in urban residents 36.8% (95% CI: 33.5–40.2%) (19). Over one-third of urban residents and university students are severely stressed. This provides further evidence that mental stress has become a serious social and public issue in China. Numerous studies have also shown an escalation of stress-related health problems (30, 31).

Addressing a gap in the literature, this study found that regional air pollution is associated with students' mental stress. Based on running full analysis models, university students that reside in higher air pollution cities had 6.67 times the likelihood of suffering from severe mental stress, compared to those who reside in lower air pollution cities. This finding is consistent with those from other studies (14–16). This indicates that mental stress is pervasive in Chinese life as is exposure to high levels of pollution. This association can also be explained by SRM model, which offers a window into the possible mechanisms that may link overuse of air pollution and health problems though mental stress (12, 13). A certain amount of stress is generally regarded as motivational, stimulating and life enhancing. Severe and persistent stress, however, may induce psychiatric disorders, other health problems, and adversely influence quality of life (32). The relationship found in this study between pollution levels and mental stress would indicate pollution to be a severe and persistent stressor, which may lead to psychiatric and health disorders. Our study results reinforces findings from prior literature (14–16) that regional air pollution is a stable variable to estimate people's exposure to air pollution.

Air pollution not only causes ill effects to the lungs and respiratory systems of the Chinese people, but it also causes stress and mental disorders, which have their own set of negative health outcomes. Air pollution is not confined to China, but is a problem in many developing countries (1). This study provides direct evidence of potential stress related health hazards due to air pollution. Since 1978, the Chinese economy has grown substantially. With the rapid development of the economy, the environment has been impacted in a negative manner. In recent years, there has been a tension between economic growth and environmental protection. Although policies of environmental protection have been adopted, they have not been strictly enforced (33). The Central government should consider adopting even more stringent environmental protection policies, while simultaneously educating the public about environmental protection and implementing environmental protection technology measures.

Some studies have hypothesized that mental stress is an intermediate variable between exposure to air pollution and health problems (8–10). More studies need to be done to determine the extent to which air pollution may be associated with the risk of developing affective psychiatric disorders. Our study suggests that air pollution is related to mental stress, which provides support for further exploration of this mechanism. While the results from this study are valuable, further studies need to be conducted.

Different from previous studies (34–36), this study that found only a individual-level variable, grade, was significantly associated with mental stress, but more environmental and regional variables were included in the associations. This indicates that environment may be more important to university students' mental stress and health problems than other individual factors. To promote student health, it is important to improve the environment that the student resides in. This study revealed that students enrolled in higher level universities exhibit lower mental stress prevalence than students in lower level universities. It is plausible that higher level universities attract outstanding students, and provide better learning resources and facilities, which results in students exhibiting less mental stress than those in lower level universities. This study also found city population where universities were located was associated with students' stress levels. Those living in larger cities had lower stress levels than those living in smaller cities. Large cities usually have more financial resources and technology, as well as better social services (25). Access to such resources may have a moderating effect on stress.

## Conclusion

This study provided new and direct evidence of an association between regional air pollution and stress levels of college students. This may increase the potential for mental disorders and other negative health impacts. This information provides support for the development, implementation, and enforcement of effective environmental protection laws, policies, and interventions. Implementing effective public education campaigns that encourage environmentally conscious actions should also be a high priority.

## Data Availability Statement

The datasets used and/or analyzed during the current study are available from the corresponding author on reasonable request.

## Author Contributions

WZ and SP drafted the manuscript. TY conceived the study design. JF, KX, HW, and YJ conducted the data collection and survey management. RC polished the language. All authors contributed to the article and approved the submitted version.

## Conflict of Interest

The authors declare that the research was conducted in the absence of any commercial or financial relationships that could be construed as a potential conflict of interest.
